# mRNA profile provides novel insights into stress adaptation in mud crab megalopa, *Scylla paramamosain* after salinity stress

**DOI:** 10.1186/s12864-020-06965-5

**Published:** 2020-08-14

**Authors:** Yin Zhang, Qingyang Wu, Shaobin Fang, Shengkang Li, Huaiping Zheng, Yueling Zhang, Mhd Ikhwanuddin, Hongyu Ma

**Affiliations:** 1 Guangdong Provincial Key Laboratory of Marine Biotechnology, Institute of Marine Sciences, Shantou University, 243 Daxue Road, Shantou, 515063 China; 2 STU-UMT Joint Shellfish Research Laboratory, Shantou University, Shantou, 515063 China; 3 Institute of Tropical Aquaculture, Universiti Malaysia Terengganu, 21030 Kuala Terengganu, Malaysia

**Keywords:** *Scylla paramamosain*, Salinity stress, Differentially expression genes, Stress adaptation

## Abstract

**Background:**

Mud crab, *Scylla paramamosain*, a euryhaline crustacean species, mainly inhabits the Indo-Western Pacific region. Wild mud crab spawn in high-salt condition and the salinity reduced with the growth of the hatching larvae. When the larvae grow up to megalopa, they migrate back to estuaries and coasts in virtue of the flood tide, settle and recruit adult habitats and metamorphose into the crablet stage. Adult crab can even survive in a wide salinity of 0–35 ppt. To investigate the mRNA profile after salinity stress, *S. paramamosain* megalopa were exposed to different salinity seawater (low, 14 ppt; control, 25 ppt; high, 39 ppt).

**Results:**

Firstly, from the expression profiles of *Na+/K+/2Cl- cotransporter*, *chloride channel protein 2*, and *ABC transporter*, it turned out that the 24 h might be the most influenced duration in the short-term stress. We collected megalopa under different salinity for 24 h and then submitted to mRNA profiling. Totally, 57.87 Gb Clean Data were obtained. The comparative genomic analysis detected 342 differentially expressed genes (DEGs). The most significantly DEGs include *gamma-butyrobetaine dioxygenase-like*, *facilitated trehalose transporter Tret1*, *sodium/potassium-transporting ATPase subunit alpha*, *rhodanese 1-like protein*, etc. And the significantly enriched pathways were lysine degradation, choline metabolism in cancer, phospholipase D signaling pathway, Fc gamma R-mediated phagocytosis, and sphingolipid signaling pathway. The results indicate that in the short-term salinity stress, the megalopa might regulate some mechanism such as metabolism, immunity responses, osmoregulation to adapt to the alteration of the environment.

**Conclusions:**

This study represents the first genome-wide transcriptome analysis of *S. paramamosain* megalopa for studying its stress adaption mechanisms under different salinity. The results reveal numbers of genes modified by salinity stress and some important pathways, which will provide valuable resources for discovering the molecular basis of salinity stress adaptation of *S. paramamosain* larvae and further boost the understanding of the potential molecular mechanisms of salinity stress adaptation for crustacean species.

## Background

Mud crab, *Scylla paramamosain* is a euryhaline crab which can live in a wide range of salinities and mostly distribute in the Indo-Western Pacific region. It has become a fairly important economic mariculture species and popular seafood in the South-East Asian countries [50]. The mud crab aquaculture has been conducted for more than 100 years in China and over the past three decades throughout the Asia [44] and occupied an increasingly momentous role in Chinese crab species farming industry [55]. However, the production is still insufficient to meet the growing demand of the consumer market, which makes the market price of the crab consistently remaining at a high level. In addition, current mud crab mariculture mainly relies on the wild-caught for seed stock of which supply is finite and unreliable, and the expansion is impeded by the lack of access to hatchery-reared seeds [44, 83]. Mud crab spends most of its life in brackish, saltwater estuaries or mangrove forests, while mature females move to offshore for spawning and hatching larvae [40]. When the larvae grow up to megalopa, they migrate back to estuaries and coasts in virtue of the flood tide, settle and recruit adult habitats and metamorphose into the crablet stage [12]. Megalopa is considered as one of the most crucial stages along the life of the mud crab due to its long habitats shifting and extensive habitats change, and the survival rate of megalopa stage significantly affects population dynamics [12]. Megalopa also constitute a dominant phase for successful larval rearing in mud crab aquaculture, since mass mortality often happens during this stage [12], which makes it an urgent issue to further promote the development of the mud crab aquaculture industry.

Ambient changes play a considerable role in controlling the abundance and distribution of aquatic habitats, especially the estuarine environment, where is the connection of the marine and fresh environment. The variations in abiotic and biotic factors are recorded to result in metabolic, immunological and physiological alterations and trigger stress to the aquatic animals [6, 27, 28, 79]. The changes of coastal, estuaries, and the culture environment of mud crab, are led by one of the basic ambient factors, salinity, which impacts physiology of the living organisms [72]. The wild and farmed mud crabs are generally exposed to climate changes, like heavy rain and large-scale water change, which directly vary the salinity of the water. Ambient salinity also can be altered by the sea level rising because of ocean acidification, global warming, and timing and amount changes of freshwater delivery [74]. The mud crab megalopa also are exposed to salinity change during the process of migration. What counts is that the salinity is closely related to osmotic pressure which has conspicuous impacts on growth, survival, immune defenses, and respiratory metabolism of marine crustaceans [9, 10, 67, 73]. Acute salinity alterations may also increase energy utilization for osmoregulation and effect the feed intake leading to poor growth of crabs [3, 60] and induce diseases [64].

Although *S. paramamosain* can survive in a broad range of salinity of 2.6–55.5 ppt [95], abrupt changes in salinity, especially the changes exceeding the species’s tolerance, will trigger stress responses. Changes in gene expression have been considered as a dominant component for stress management [6, 49, 63, 66, 90]. Previous study on crab larvae showed that the survival rate could be significantly affected by acute salinity changes [65]. When ambient salinity changes, the mud crab megalopa might compensate for ion loss or excess via osmoregulation by burning more energy, along with a series of stress-induced adaptions, which was the goal of the present study to uncover the molecular alterations caused by salinity adaptions. In recent years, molecular approaches such as microarray, gene library construction, gene expression profiling, transcriptome analysis, and next-generation sequencing have accelerated the search for genes and genetic pathways that respond to environmental challenges by changing their expression, which provides a basic tool for detection of the molecular mechanisms involved in these responses [77, 90]. Studies have investigated the cellular and molecular responses of osmoregulatory enzymes and ion-transport proteins in crustaceans under drastic and long-term salinity stress [13, 17, 37–39]. However, the molecular responses under different levels of salinity have rarely been reported in *S. paramamosain*, let alone megalopa. Xu and Liu [90] previously reported transcriptome analysis of adult swimming crab *Portunus trituberculatus* under salinity stress, revealing the expression patterns under low and high salinity conditions compared with optimal salinity to absorb the salinity stress and recognize osmoregulation-related genes of the swimming crab. Hui et al. [42] performed RNA sequencing of Chinese mitten crab, *Eriocheir sinensis* megalopa before and after desalination indicated that the megalopa might cause a series of stress-induced genes and pathways under low salinity environment. Few information is gainable related to the alterations in genes expressions related to salinity tolerance in *S. paramamosain* megalopa. *S. paramamosain* can provide a good model organism, from the perspective of its representative euryhaline habitat and migration life history as well as its important economic value [82], for future studies on physiological, biochemical and immune gene regulation pathways associated with different environmental variations, and also indicators of salinity changes in the offshore environment.

The Na+/K+/2Cl- cotransporter (NKCC), an integral membrane protein, functioned in transporting Na^+^, K^+^ and 2Cl^−^ into cells and plays an indispensable role in osmotic regulation and cell ionic [25, 26]. The expression of *NKCC* is sensitive to salinity, and its transcription level is significantly influenced by salinity in crustaceans [36, 57]. Chloride channel protein 2 (ClC2) is a ubiquitously expressed chloride channel [78] and can be activated by hypotonic swelling [32]. ABC transporters (ABCs) constitute one of the largest protein families that transport different set of substrates across biological membranes including amino acids, ions, toxic metabolites, sugars, xenobiotics, and polypeptides [16, 41], and play vital roles in protecting organisms from the varied environment [56]. To initially detect the impacts of different salinity on the of *S. paramamosain* megalopa in molecular level, we monitor the time-dependent (0 h, 12 h, 24 h, 48 h and 72 h) expressions of *NKCC*, *ClC2* and *ABC* genes in megalopa under different salinity treatments. Herein, according to the expression profile of the three osmoregulation related genes, megalopa under salinity stress after certain time will be subjected to high throughput transcriptome sequencing. The present study attempted to unveil the molecular basis of the stress adaption mechanisms of larvae at crucial developmental stages under high and low salinity stress with a transcriptomic analysis of *S. paramamosain* megalopa. Given the critical role in raising larvae in aquaculture, understanding the molecular level of environmental tolerance and physiological changes is of great value for its management and potential culture.

## Results

### Time-dependent expression in different salinity treatments

**Fig. 1:**
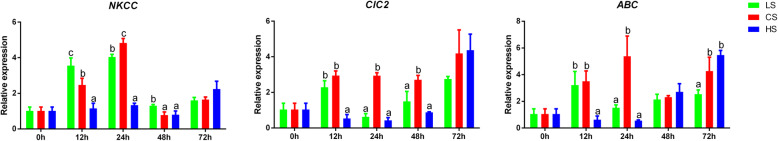
Relative expressions of genes related to ion transport (*ABC, ClC2, NKCC*) in *Scylla paramamosain* megalopa under different salinities after 0 h, 12 h, 24 h, 48 h, and 72 h. LS represents low salinity of 14 ppt, CS represents control group of 25 ppt salinity, HS represents high salinity of 39 ppt. The different letters in the table below represent the significant differences between groups (*p* < 0.05)

The experiment was carried out setting three levels of salinities, namely 14, 25 and 39 ppt. The salinity of 14 ppt was relatively low salinity (LS) while 39 ppt was high salinity (HS), and 25 ppt was set as control group (CS). To preliminary explore the molecular level alterations of osmoregulation under different salinity and period of exposure time, the expressions of osmoregulation related genes *NKCC*, *ClC2* and *ABC*, which were closely related to the osmotic changes, were detected under low and high salinity after 0 h, 12 h, 24 h, 48 h and 72 h (Fig. 1). The osmoregulation related gene *NKCC* was significantly down-regulated at 24 h under high salinity. The ion transport-related genes *ClC2* and *ABC* were significantly down-regulated both at 12 h and 24 h under high salinity. Surprisingly, genes showed no significant differences after 48 h and 72 h under salinity alterations, especially at 48 h the expressions were nearly the same in the three groups and a little deviation presented at 72 h between the three levels of salinity treatment. Taken together, we chose to further study the gene expressions of the megalopa under low and high salinity after 24 h, thus the 24 h treatment samples were collected and subjected to the transcriptome sequencing.

### Sequencing and assembly and functional annotation

To detect the effects of different salinity on *S. paramamosain* megalopa, next-generation sequencing was used to explore the variation of differentially expressed genes. After filtering on raw data, removing adaptor and low-quality reads, 57.87 Gb Clean Data was obtained from LS, CS and HS group in total using Sequencing By Synthesis (SBS) technic. The minimum of base score Q30 was over 92.79% (supplementary Table 2). Subsequently, the clean data were subjected to map with the reference genome (unpublished data) with mapping ratio from 75.65 to 79.14%. All transcriptome data are available in the NCBI Short Read Archive (SRA) database under accession SRR10493620, SRR10493619 and SRR10493618 corresponding to the LS, CS and HS group, respectively.

A total of 22,766 genes were successfully annotated by aligning to the databases NR, Swiss-Prot, Pfam, KOG, GO and KEGG. For NR species distribution, 21,961 genes matched known sequence from 448 species. Most genes hits were to *Hyalella Azteca* (6659 genes, 30.40%), *Cryptotermes secundus* (952 genes, 4.35%), *Zootermopsis nevadensis* (571 genes, 2.61%), *S. paramamosain* (539 genes, 2.46%), *Apostichopus japonicus* (477 genes, 2.18%) (supplementary Fig 1). Besides, 11,092 and 14,907 genes were annotated against the protein databases Swiss-Prot and Pfam, respectively.

As a consequence, 13,661 genes were annotated in KOG and grouped into 25 KOG classifications (supplementary Fig 2). The largest cluster was “the general function prediction only (R)”, indicating the functions of most genes were predicted by informatics methods and has yet to be confirmed by experimentation, and then followed by “signal transduction mechanisms (T)” and “posttranslational modification, protein turnover, chaperones (O)”.

Through the alignment of GO databases, 8468 genes were annotated to 53 terms of GO classification (supplementary Fig 3). For Cellular Component, cell part (2987 genes), cell (2979 genes), membrane, (2440 genes), organelle (1876 genes), membrane part (1431 genes) represented the majority category of this category. Catalytic activity (4009 genes), binding (3743 genes), transporter activity (644 genes), structural molecule activity (305 genes), signal transducer activity (264 genes) represented the majorities of Molecular Function. Cellular process (3756 genes), metabolic process (3515 genes), single-organism process (2242 genes), biological regulation (1503 genes), localization (1232 genes) represented a high percentage of the Biological Process category, followed by response to stimulus (836 genes) which drew the attention.

Besides, 9877 genes were enriched to 280 KEGG pathways. The pathways including purine metabolism (199 genes), endocytosis (190 genes), lysosome (179 genes), RNA transport (177 genes), protein processing in endoplasmic reticulum (169 genes), phagosome (167 genes), spliceosome (164 genes), ubiquitin mediated proteolysis (160 genes), ribosome (152 genes) and carbon metabolism (134 genes) occupied the Top 10 pathways that enriched the most genes.

### Differentially expressed genes and functional annotation

**Fig. 2:**
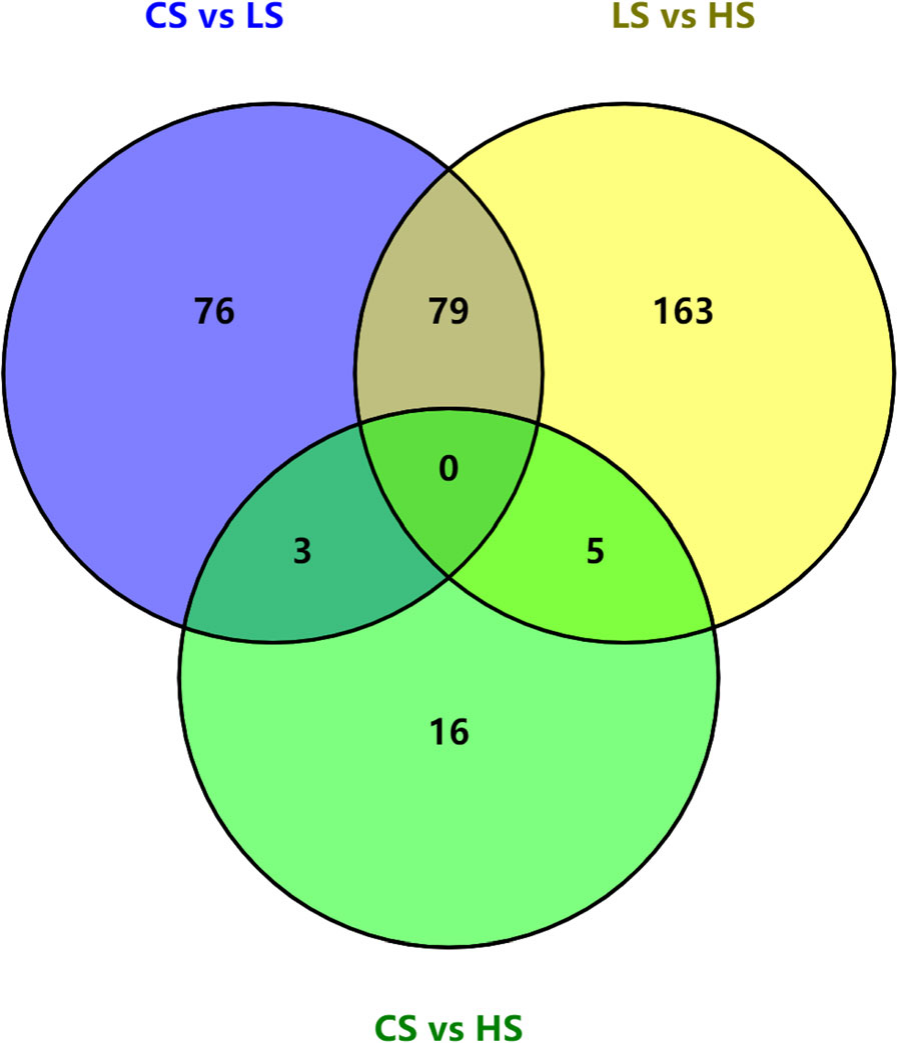
A venn diagram showing the number of differentially expressed genes between pairwise comparisons of *Scylla paramamosain* megalopa under different salinities. LS represents low salinity of 14 ppt, CS represents control group of 25 ppt salinity, HS represents high salinity of 39 ppt

According to the expressions of the genes in LS, CS, and HS groups, the DEGs were detected in pairwise comparisons. Together, 342 DEGs were spotted, sharing no common DEGs between the three comparisons (Fig. 2). In the comparison of CS vs LS group, 158 DEGs with 87 up-regulated and 71 down-regulated were found (Table 1, supplementary Table 3).

**Table 1:** The summary of differentially expressed genes

Treatment comparisons	DEG Number	Up-regulated	Down-regulated
CS vs LS	158	87	71
LS vs HS	247	120	127
CS vs HS	24	11	13

*LS* Represents low salinity of 14 ppt, *CS* Represents control group of 25 ppt salinity, *HS* Represents high salinity of 39 ppt, *DEG* Represents differentially expressed gene

**Fig. 3:**
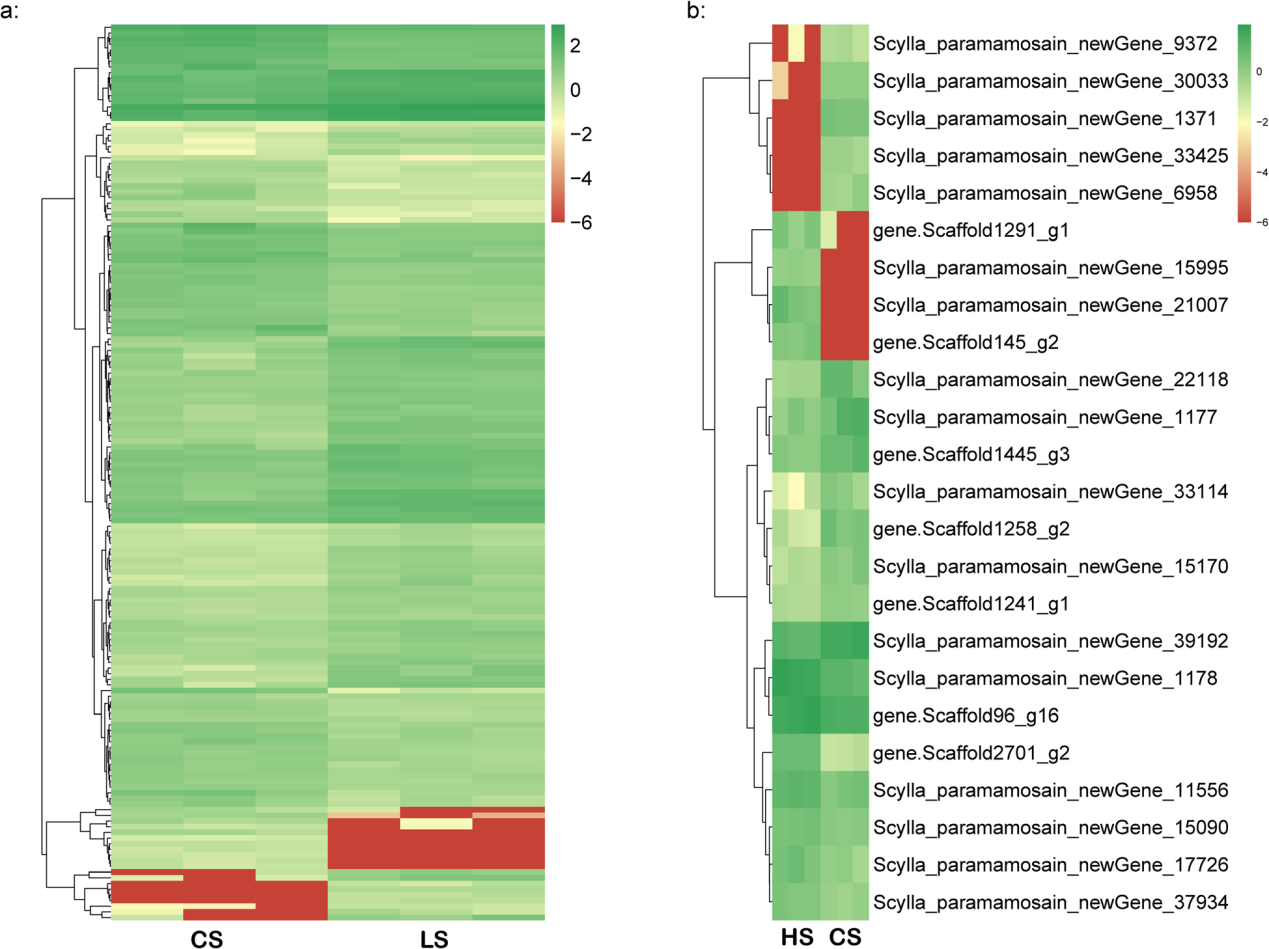
Heatmap of the differentially expressed genes in the comparison of CS vs LS (**a**) and CS vs HS (**b**). LS represents low salinity of 14 ppt, CS represents control group of 25 ppt salinity, HS represents high salinity of 39 ppt

**Fig. 4:**
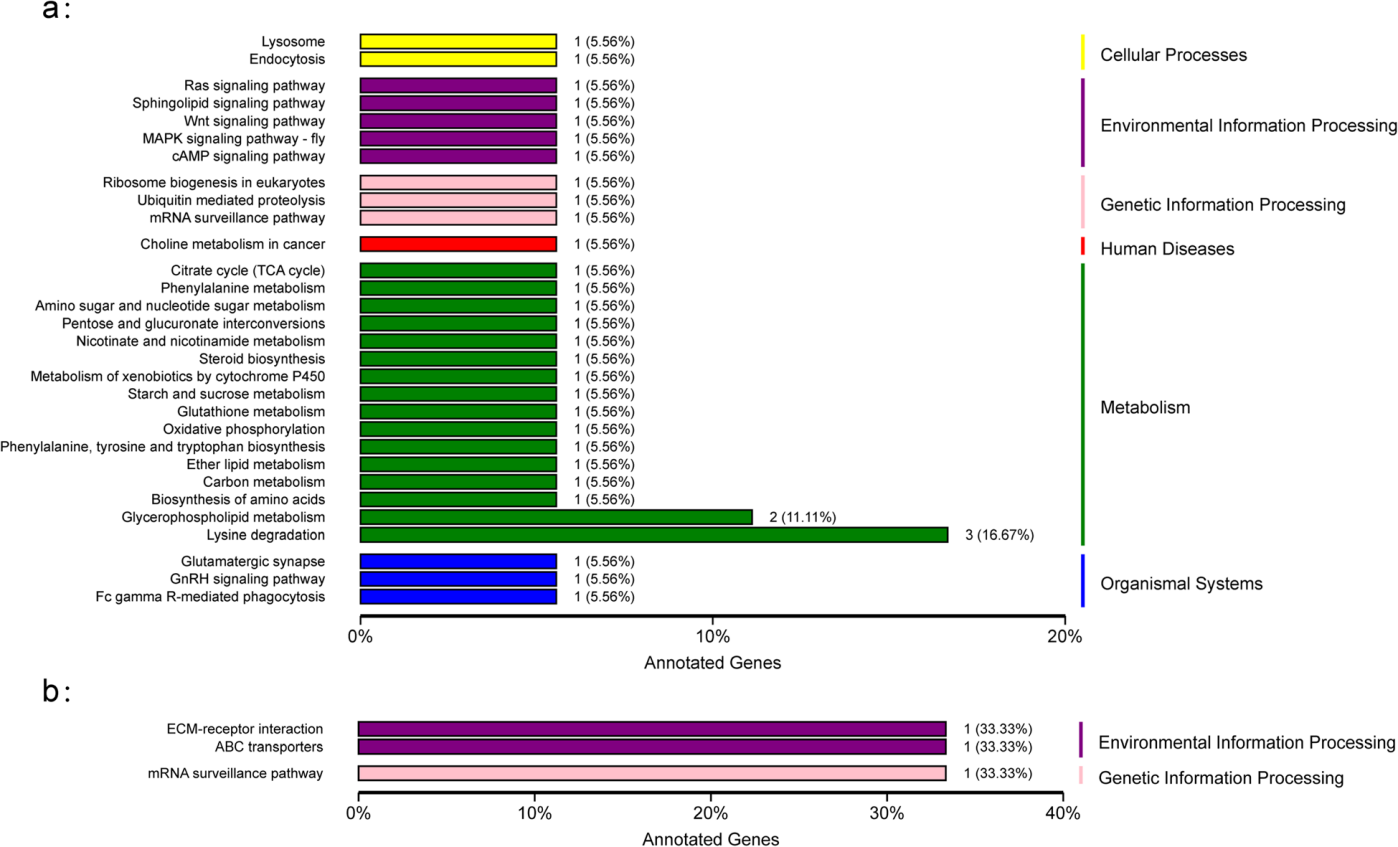
KEGG pathways enrichment of the differentially expressed genes in the comparison of CS vs LS (**a**) and CS vs HS (**b**). LS represents low salinity of 14 ppt, CS represents control group of 25 ppt salinity, HS represents high salinity of 39 ppt

In the CS vs LS comparison, the most significantly differentially expressed genes including *gamma-butyrobetaine dioxygenase-like* (*GBDL*), *facilitated trehalose transporter Tret1* (*TRET1*), *sodium/potassium-transporting ATPase subunit alpha* (*NKTASα*), *rhodanese 1-like protein* (*RHD1-like*), *proton-coupled folate transporter-like* (*PCFTL*), *estrogen sulfotransferase* (*ESULT*), *hemocyanin subunit 2* (*HcS2*), *choline transporter-like protein 1* (*CTL1*), *NKCC*, *vitellogenin-like* (*VTGL*) which were most significantly up-regulated in LS group, and *casein kinase II subunit alpha* (*CK2α*), *calponin/transgelin*, *zinc finger RNA-binding protein-like* (*ZFRBL*), *aminopeptidase N-like* (*APNL*), *mucin-1*, *neprilysin-11-like* (*NEP11L*), *microtubule-associated protein 1A-like* (*MAP 1AL*) which were most significantly down-regulated in LS group (Table 2; Fig. 3a). The DEGs were further subjected to GO annotations to predict their potential functions, and KEGG enrichment to obtain relevant metabolic pathways. Table 3 shows the GO classifications of the DEGs in comparisons of LS and CS group. Oxidation-reduction process, chloride transport, cell adhesion, tube development and ion transmembrane transport were the most significantly enriched GO function items in the category of Biological Process. In the Cellular Component, integral component of membrane, membrane, extracellular space, plasma membrane and cell junction were the most significant items, while metal ion binding, “oxidoreductase activity, acting on paired donors, with incorporation or reduction of molecular oxygen”, iron ion binding, voltage-gated chloride channel activity and endopeptidase activity were the most significant terms in Molecular Function. The top five significantly enriched KEGG pathways were lysine degradation, choline metabolism in cancer, phospholipase D signaling pathway, Fc gamma R-mediated phagocytosis and sphingolipid signaling pathway (Fig. 4a).

**Table 2:** Top differentially expressed genes in pairwise comparisons of *Scylla paramamosain* megalopa

#Gene ID	FDR	log2FC	Regulated	Gene name
**CS vs LS**				
gene.Scaffold1859_g1	1.18E-26	3.290026	up	gamma-butyrobetaine dioxygenase-like
Scylla_paramamosain_newGene_6838	1.47E-16	2.989931	up	gamma-butyrobetaine dioxygenase-like
Scylla_paramamosain_newGene_6835	5.13E-33	2.44134	up	gamma-butyrobetaine dioxygenase-like
Scylla_paramamosain_newGene_33600	2.59E-10	2.335425	up	facilitated trehalose transporter Tret1
gene.Scaffold900_g16	1.14E-19	2.333814	up	putative defense protein 3
gene.Scaffold728_g5	5.51E-15	2.212924	up	sodium/potassium-transporting ATPase subunit alpha
gene.Scaffold1064_g3	2.38E-38	2.129319	up	rhodanese 1-like protein
Scylla_paramamosain_newGene_6834	4.13E-15	2.082766	up	gamma-butyrobetaine dioxygenase-like
Scylla_paramamosain_newGene_16509	4.33E-07	2.01342	up	retrovirus-related Pol polyprotein from type-1 retrotransposable element R1
Scylla_paramamosain_newGene_33599	4.42E-07	1.97739	up	facilitated trehalose transporter Tret1-like
Scylla_paramamosain_newGene_29537	2.34E-12	1.958022	up	hypothetical protein DAPPUDRAFT_316225
Scylla_paramamosain_newGene_30171	2.00E-07	1.881975	up	proton-coupled folate transporter-like
gene.Scaffold3248_g1	6.40E-06	1.821815	up	estrogen sulfotransferase
Scylla_paramamosain_newGene_1164	1.90E-10	1.57869	up	uncharacterized protein LOC108678138
Scylla_paramamosain_newGene_24744	2.06E-11	1.532331	up	uncharacterized protein LOC108665683
gene.Scaffold454_g9	1.97E-05	1.409072	up	uncharacterized protein LOC108679078
gene.Scaffold2007_g4	0.00127	1.376571	up	hemocyanin subunit 2
gene.Scaffold928_g5	0.000491	1.355006	up	choline transporter-like protein 1
gene.Scaffold1510_g2	2.93E-10	1.341927	up	Na+/K+/2Cl- cotransporter
gene.Scaffold286_g11	0.005894	1.326881	up	vitellogenin-like protein
Scylla_paramamosain_newGene_8114	7.36E-35	−4.11054	down	casein kinase II subunit alpha, partial
Scylla_paramamosain_newGene_10699	4.57E-30	−3.6122	down	calponin/transgelin
gene.Scaffold1258_g2	1.41E-07	−2.09292	down	zinc finger RNA-binding protein-like isoform X3
Scylla_paramamosain_newGene_32270	3.42E-06	−1.88317	down	hypothetical protein OCBIM_22027343mg, partial
Scylla_paramamosain_newGene_19291	7.58E-13	−1.85211	down	aminopeptidase N-like
Scylla_paramamosain_newGene_27140	8.06E-06	−1.81576	down	mucin-1
gene.Scaffold3610_g1	5.05E-09	−1.66044	down	neprilysin-11-like
gene.Scaffold271_g7	2.08E-06	−1.61464	down	gamma-butyrobetaine dioxygenase-like
Scylla_paramamosain_newGene_40084	3.64E-06	−1.60139	down	microtubule-associated protein 1A-like
gene.Scaffold132_g13	0.00018	−1.58708	down	uncharacterized protein APZ42_025850
Scylla_paramamosain_newGene_29679	0.000254	−1.56656	down	hypothetical protein KP79_PYT22069
Scylla_paramamosain_newGene_36343	0.000281	−1.56571	down	proton-coupled folate transporter-like
Scylla_paramamosain_newGene_21183	0.000516	−1.49135	down	conserved Plasmodium membrane protein, unknown function
Scylla_paramamosain_newGene_42681	8.73E-06	−1.46576	down	cuticle protein AM
Scylla_paramamosain_newGene_25183	0.000497	−1.4642	down	reverse transcriptase, partial
Scylla_paramamosain_newGene_13981	0.001003	−1.43755	down	Succinate dehydrogenase [ubiquinone] flavoprotein subunit, mitochondrial
gene.Scaffold1359_g15	0.005213	−1.34713	down	(E3-independent) E2 ubiquitin-conjugating enzyme UBE2O-like isoform X1
Scylla_paramamosain_newGene_34432	0.004251	−1.34333	down	clotting protein precursor, partial
Scylla_paramamosain_newGene_25256	0.001576	−1.28034	down	peritrophin-44-like protein
**CS vs HS**				
gene.Scaffold96_g16	0.004113	1.046966	up	projectin
gene.Scaffold1291_g1	0.002581	1.121082	up	UPF0183 protein CG7083 isoform X2
gene.Scaffold145_g2	0.002359	1.13704	up	ubiA prenyltransferase domain-containing protein 1 homolog
Scylla_paramamosain_newGene_21007	0.001607	1.164676	up	basement membrane-specific heparan sulfate proteoglycan core protein, partial
Scylla_paramamosain_newGene_11556	0.000132	1.396239	up	multidrug resistance-associated protein 1 isoform X1
gene.Scaffold1258_g2	2.18E-08	−1.79188	down	zinc finger RNA-binding protein-like isoform X3
Scylla_paramamosain_newGene_39192	0.000382	−1.27846	down	gamma-interferon induced thiol reductase GILT3
gene.Scaffold1445_g3	0.003801	−1.18908	down	structural maintenance of chromosomes protein 2-like
gene.Scaffold1241_g1	0.002581	−1.16992	down	uncharacterized protein LOC108666206

*LS* Represents low salinity of 14 ppt, *CS* Represents control group of 25 ppt salinity, *HS* Represents high salinity of 39 ppt

**Table 3:** Top 5 GO annotations of the three categories (BP: Biological Process, CC: Cellular Components, MF: Molecular Function) significantly enriched by differentially expressed genes

	GO ID	Term	Annotated	Significant	Expected	*P*-value
**CS vs LS**						
BP	GO:0055114	oxidation-reduction process	413	4	1.73	0.00017
BP	GO:0006821	chloride transport	14	4	0.06	0.00047
BP	GO:0007155	cell adhesion	75	1	0.31	0.00089
BP	GO:0035295	tube development	10	1	0.04	0.00584
BP	GO:0034220	ion transmembrane transport	250	2	1.05	0.00624
CC	GO:0016021	integral component of membrane	1230	9	4.39	4.2E-06
CC	GO:0016020	membrane	2483	11	8.87	0.0043
CC	GO:0005615	extracellular space	51	1	0.18	0.0125
CC	GO:0005886	plasma membrane	325	3	1.16	0.0304
CC	GO:0030054	cell junction	66	2	0.24	0.033
MF	GO:0046872	metal ion binding	1001	4	4.91	0.00079
MF	GO:0016705	oxidoreductase activity, acting on paired donors, with incorporation or reduction of molecular oxygen	70	1	0.34	0.00107
MF	GO:0005506	iron ion binding	50	1	0.25	0.00128
MF	GO:0005247	voltage-gated chloride channel activity	4	2	0.02	0.00165
MF	GO:0004175	endopeptidase activity	188	1	0.92	0.00337
**CS vs HS**						
BP	GO:0051276	chromosome organization	156	1	0.05	0.02219
BP	GO:0006996	organelle organization	427	1	0.15	0.48991
BP	GO:0006793	phosphorus metabolic process	815	1	0.28	0.76061
BP	GO:0009987	cellular process	4286	2	1.49	0.8504
BP	GO:0006796	phosphate-containing compound metabolic process	799	1	0.28	0.85274
CC	GO:0016021	integral component of membrane	1230	1	0.52	0.000016
CC	GO:0016020	membrane	2483	1	1.04	0.0039
CC	GO:0031224	intrinsic component of membrane	1235	1	0.52	0.1317
CC	GO:0044464	cell part	2983	1	1.25	0.7094
CC	GO:0005623	cell	3005	1	1.26	0.7295
MF	GO:0046872	metal ion binding	1001	1	0.45	0.00059
MF	GO:0043169	cation binding	1018	1	0.45	0.47576
MF	GO:0004659	prenyltransferase activity	10	1	0	0.49112
MF	GO:0016765	transferase activity, transferring alkyl or aryl (other than methyl) groups	27	1	0.01	0.58447
MF	GO:0005488	binding	3747	2	1.67	0.69304

*LS* Represents low salinity of 14 ppt, *CS* Represents control group of 25 ppt salinity, *HS* Represents high salinity of 39 ppt

In the comparison of CS vs HS group, 24 DEGs were found, of which 11 were up-regulated and 13 were down-regulated in the HS group (Table 1, supplementary Table 4). The up-regulated genes included *multidrug resistance-associated protein 1 isoform X1* (*MRP1*), *ubiA prenyltransferase domain-containing protein 1 homolog*, *projectin* (*PRJ*) and down-regulated genes such as *zinc finger RNA-binding protein-like isoform X3 (ZFRB)*, *structural maintenance of chromosomes protein 2-like (SMC2)* and *gamma-interferon induced thiol reductase GILT3* (*GILT3*) under high salinity condition (Table 2; Fig. 3b). The GO annotation only covered four DEGs, resulting in 25 GO terms (Table 3). Phosphorus metabolic process, phosphate-containing compound metabolic process, cellular process, metabolic process and cellular metabolic process were the most significant GO items in the category of Biological Process. As to the Cellular Components category, the DEGs were enriched for five items including integral component of membrane, membrane, intrinsic component of membrane, membrane part, cellular component. Prenyltransferase activity, transferase activity, transferring alkyl or aryl (other than methyl) groups, heterocyclic compound binding, organic cyclic compound binding and binding were the top five significantly abundant items in Molecular Function. Only 3 pathways were significant in the KEGG pathway enrichment tests, namely ECM-receptor interaction, ABC transporters and mRNA surveillance pathway (Fig. 4b).

### Validation of RNA-seq profile results by qRT-PCR

**Fig. 5:**
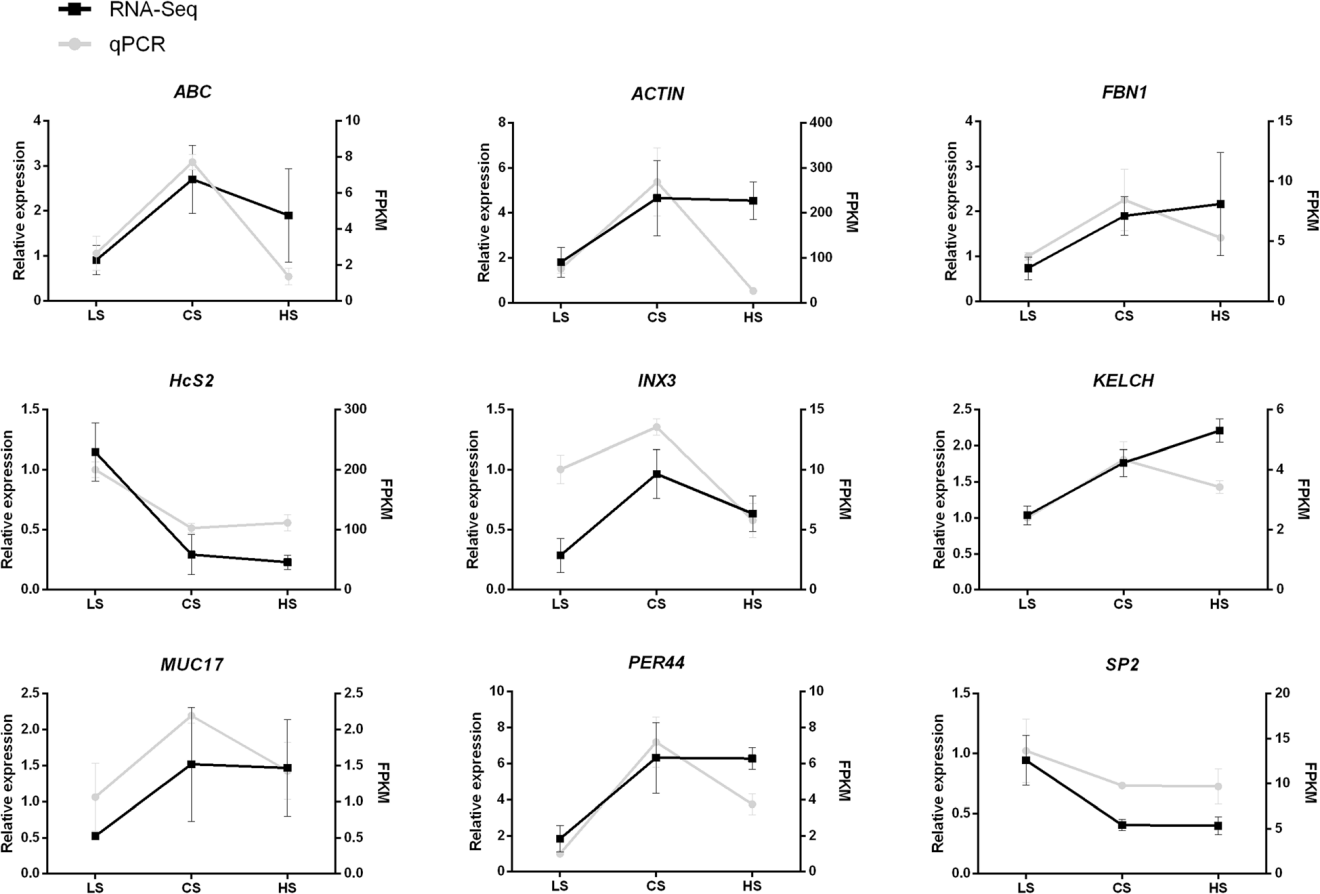
Candidate gene expression levels revealed by qRT-PCR and RNA-seq. LS represents low salinity of 14 ppt, CS represents control group of 25 ppt salinity, HS represents high salinity of 39 ppt

To validate the RNA sequencing, nine genes were selected among the Top 50 DEGs in LS and HS groups and quantified using qRT-PCR, namely *ABC transporter* (*ABC*), *actin (ACTIN)*, *fibrillin-1-like* (*FBN1*), *HcS2*, *innexin 3* (*INX3*), *kelch-like ECH-associated protein 1 (KELCH)*, *mucin-17-like (MUC17)*, *peritrophin-44-like protein (PER44)* and *serine protease 2 (SP2)*. The relative expression tendencies of the nine genes were in accordance with the RNA-seq (Fig. 5), validating the results of the mRNA sequencing analysis.

## Discussion

### Alterations of osmoregulation and ion transport-related genes under salinity stress during different duration

According to the initial detection, the osmoregulation and ion transport-related genes *NKCC, ClC2* and *ABC* were significantly down-regulated under low salinity and high salinity after 24 h. As it’s known that NKCC functions in transporting Na^+^, K^+^ and 2Cl^−^ into cells and plays an essential role in osmotic regulation and cell ionic [25, 26]. The transcription level of NKCC is significantly influenced by salinity in crustaceans [36, 57]. The expression change of *NKCC* in a euryhaline crab *Chasmagnathus granulatus* against higher and lower salinity seawater transfer revealed NKCC-driven salt secretion and ion uptake [57]. C1C2 is a ubiquitously expressed chloride channel [78]. It can be activated by hypotonic swelling and may function in cell volume regulation [32]. ABC protein is involved in the regulation of membrane ion channels, especially ATP-sensitive potassium channels [52], and the *ABC* gene differentially expressed in the present study is most likely to be involved in osmoregulation. The expression of important ion transport-related genes was down-regulated to prevent the high concentration of ions in the environment from penetrating into the body during short-term salinity stress. Besides, the functional processes like chloride transport, metal ion binding and iron ion binding which were mostly related to ion transport were significantly enriched by DEGs. The results showed that low salinity affects the activity of ion channel [71], which is closely related to the osmotic regulation of *S. paramamosain*.

Dilution or elevation of salinity appears to trigger a classic “stress response” at the level of transcription in *S. paramamosain*. Strongest differentially expression response in crustaceans tends to occur 1–3 days after salinity transfer [35]. Together with our preliminary detection of osmoregulation related genes under different salinity and period of exposure time, the alterations were obvious after 24 h. Thus, we choose megalopa under salinity stress for 24 h to identify the genes expressions changes. Hundreds of DEGs were obtained in the present study. Totally, 342 DEGs were obtained including 158 DEGs in the comparison of LS group and CS group, 24 DEGs in HS vs CS and 247 DEGs in LS vs HS. More DEGs were detected in low salinity treatment than that in high salinity, which might be related to the living habitat of megalopa. The observation of fewer DEGs at high salinity suggests that the megalopa could acclimatize themselves to the high salinity of 39 ppt in the current study and might be sensitive to low salinity. Before the zoea metamorphosed to megalopa, they lived in a relatively high salinity environment of which the salinity reached 35 ppt [58]. Among the DEGs, some genes that were most differentially expressed in low or high salinity might play critical roles in adapting the changes of the salinity environment. The differences in gene expressions may mirror the adaptation mechanism of the mud crab megalopa how the larvae survive under salinity condition changes. To identify the responses to changes in salinity at the molecular level, comparisons of gene expressions among the different treatment groups in the present experiment could facilitate the identification of candidate genes underlying response to salinity stress in *S. paramamosain* larvae.

### DEGs under salinity stress indicated stress adaptation of crab larvae

The most significantly up-regulated genes in LS group includes up-regulated genes related to metabolism (*GBDL*, *PCFT*, *ESULT*, *RDL1*), osmoregulation (*NKTASα*, *CTL1*, *NKCC*) and immunity (*TRET1*, *HcS2*, *VTGL*). GBD enzyme is required for carnitine synthesis which is an essential role in fat metabolism [86]. GBDL belongs to the lysine metabolism pathway in the present study. PCFT was regarded as the molecular factor of the carrier-mediated intestinal folate transport system [15, 68]. ESULT was identified as an essential role by adding a sulfate group to estradiol, subsequently it dissolved in the blood and hemolymph to circulate throughout the body [14, 30]. The RDL proteins have roles in “managing” stress tolerance and in maintenance of redox homeostasis [70, 76]. The knockout of rhodanese genes (*RDL1* and *RDL2*) made a wine yeast strain *Saccharomyces cerevisiae* more sensitive to oxidative stress [62]. The up-regulation of *GBDL*, *PCFT, ESULT* and *RDL1* genes in LS group implied that the megalopa might attempt to adapt the short-term change by adjusting themselves by regulation of homeostasis.

It is no surprise that the osmoregulation related genes including *NKTASα*, *CTL1* and *NKCC* were upregulated under the low salinity condition. The Na+/K + -ATPase is a crucial enzyme to the homeostasis of cell volume, osmotic pressure and the maintenance of electrochemical gradients [2]. CTL1, is involved in supplying choline to certain cell types [61], and the uptake of choline is weakly Na^+^ dependent [24]. What caught our attention was that the most significantly DEGs containing *TRET1*, *HcS2* and *VTGL* which were directly or indirectly linked to the immunity stress were also up-regulated in LS group. The TRET1 is a trehalose-specific facilitated transporter, and trehalose may be a useful cryoprotective or dehydrating molecule for cells and biological molecules such as proteins and nucleotides [45]. *Tret1* expression was also induced by salinity stress in an anhydrobiotic insect [45]. Besides, *HcS2* was up-regulated under low salinity. Hemocyanin is responsible for oxygen transport [18] which might be affected by the salinity change here. Also, hemocyanin plays an important role in non-specific innate immune defense and is an effective immune defense molecule in arthropods [18]. The hemocyanin subunit is the functional group of crustacean immunity. The expression of hemocyanin subunits varies with environmental or nutritional changes in lobsters [7]. It was reported that HcS2 helped the processed hemocyanin exhibit phenoloxidase activity in crayfish [51]. Vitellogenin is known to be associated with oocyte and embryo development in crab species [91]. Surprisingly, *VTGL* was up-regulated when the *S. paramamosain* megalopa were exposed to low salinity. We speculated that the up-regulated vitellogenin in the present study might play an immune-relevant role, as previous studies also showed that vitellogenin functioned part in immunity. The first solid evidence of vitellogenin performing an immune-relevant role was observed after vitellogenin was purified from the ovaries of *Protochordate amphioxus*, *Branchiostoma japonicum*. It has agglutination activity to chicken, toad and grass carp erythrocytes and antibacterial activity to Gram-negative bacterium, *Escherichia coli* [94]. After that, vitellogenin from the scallop, *Mizuhopecten yessoensis* was also shown to have antibacterial activity [87] as well as some other vertebrates and invertebrates [75].

The top significantly down-regulated genes were mainly related to cell differentiation (*CK2α*, *calponin/transgelin*, *ZFRBL*, *MAP 1AL*) and immunity (*APNL*, *mucin-1*, *NEP11L*). CK2 is a serine/threonine-selective protein kinase involved in DNA repair, regulation of the circadian rhythm, cell cycle control, and other cellular processes [1, 33]. CK2 was reported to be implicated in phosphorylation of MAP 1B, together with MAP 1A, during neuroblastoma cell differentiation [21]. Calponins and transgelins are members of a conservative family of actin related proteins that are widely expressed from yeast to humans [29]. Increased levels of transgelin expression have been associated in cell differentiation and senescence [31]. ZFRBL is responsible for direct, high-affinity binding to messenger RNA (mRNA). The hypotonic environment might reduce the growth from the down-regulation of *CK2α*, *calponin/transgelin*, *ZFRB* and *MAP 1AL* which were related to the cell differentiation, thus leading to slowly metamorphosis. The delay of metamorphosis under salinity stress was found in the megalopa of euryhaline crabs *Armases miersii* [3] and mud crab, *Scylla serrata* [60]. The deferred growth under inapposite salinity conditions might be out of the alteration of homeostasis responding to the stress [22]. Aminopeptidase N (APN) is known to selectively decompose amino terminus of proteins or oligopeptides [59]. In animals, APN is most abundant in the brush border membrane of the intestines, accelerating the digestion of absorbed proteins [47]. In the insect midgut, however, APN can act as a receptor for binding to toxin proteins produced by viruses and bacteria [69], which might be its responsibility in the present study. Mucin is the main component of infection-fighting mucous secreted by goblet cells in the intestine [20]. NEP11L was related to neuroendocrine system and might be involved in immune mechanisms [85]. The expression alterations of *APNL*, *mucin-1* and *NEP11L* genes here demonstrated that the salinity variation of the environment might cause immunity responses of the larvae.

The most significantly expressed genes in HS group turned out to be up-regulated genes including *MRP1*, *PRJ* and down-regulated genes such as *ZFRB*, *SMC2* and *GILT3*, which were mainly implicated in immunity and metabolism. The MRP1 is a major active transporter of glucuronate, glutathione, and sulfate conjugates [53] but can also transport unconjugated xenobiotics [93]. It was up-regulated in HS, indicating that high salinity might play as xenobiotics triggering the defense of the body to maintain its homeostasis. PRJ plays a crucial role in stretch activation [81]. It might function in the development of the abdominal stretch of the megalopa whose abdominal will retract under its carapace during this stage. The down-regulated gene ZFRB is responsible in biding mRNA. The main function of GILT is to promote the full expansion of lysosomal degraded proteins by releasing the structural constraints imposed by in-chain and interchain disulfide bonds [5]. SMC proteins are found in almost all organisms. Members of SMC protein family are involved in chromosome coagulation and sister chromatid cohesion and in most cases essential for viability [34].

The most significant DEGs in the low salinity or high salinity were mainly involved in mechanisms such as metabolism, cell differentiation, immunity and osmoregulation. Thus, the megalopa possibly maintain its homeostasis against the environment changes through regulating those DEGs. It indicated that the megalopa as a crab larvae has the ability to protect itself from the short-term salinity stress, which might be closely related to its life history of migration.

### Functional enrichment of DEGs showed homeostasis regulation after salinity stress

The oxidation-reduction process, a GO functional item that is significantly enriched by DEGs in LS group, is crucial for the maintenance of cell homeostasis. Under physiological conditions, cells maintain the oxidation-reduction balance through the production and elimination of reactive oxygen /nitrogen species (ROS/RNS) [80]. Normally, homeostasis during redox ensures that cells respond appropriately to both endogenous and exogenous stimuli. However, when the homeostasis of the redox process is disrupted, oxidative stress can lead to abnormal cell death and promote the development of disease [80]. Both exogenous and endogenous contribute to the formation of intracellular ROS/RNS. This study suggested that exposure to salinity stress might activate the oxidation-reduction process in *S. paramamosain* megalopa thus balancing the level of ROS/RNS. Of course, it is also possible that *S. paramamosain* was disturbed in the oxidation-reduction process under salinity stress.

This study revealed several important salinity adaptation pathways, which is helpful to understand the molecular basis of osmotic regulation and salinity adaptation in mud crabs. The top five significantly enriched KEGG pathway by DEGs in LS were lysine degradation, choline metabolism in cancer, phospholipase D signaling pathway, Fc gamma R-mediated phagocytosis and sphingolipid signaling pathway, while only three KEGG pathways (ECM-receptor interaction, ABC transporters and mRNA surveillance pathway) were enriched by the DEGs in HS.

The lysine degradation pathway, enriched most DEGs, was also found to be significantly enriched under chronic low salinity stress in the Pacific white shrimp [11]. It could indirectly affect ion transfer or energy metabolism, promoting “compensation process”, which is consistent with the reports of Chinese mitten crab and Pacific white shrimp under salinity stress [11, 54]. Choline in animal tissues is the main component of the neurotransmitter acetylcholine, and function with inositol as a basic component of lecithin [84]. Besides, phospholipase D catalyzes the hydrolysis of phosphatidylcholine to phospholipid acid and choline. It acts as a regulator of intercellular signaling and metabolic pathways, especially when cells are under stress [8]. Fc gamma R-mediated phagocytosis, immunity-associated pathway, uses actin and microtubule drives to achieve phagocytic formation and intracellular transport between phagocytes and other endocytic compartments [4]. Sphingolipid signaling regulates cellular fitness through a variety of mechanisms. It was shown to be necessary for endocytosis [19, 23]. Sphingolipid signaling may constitute a programmed stress response that occurs before the evolution of apoptosis [48]. Those pathways significantly enriched by DEGs reflected that the megalopa might activate some self-protection system such as ion transfer or energy metabolism, immune response and apoptosis in the short-term salinity stress.

## Conclusions

In conclusion, we analyzed transcriptomic changes in ambient salinity challenges of three different salinities, namely LS, CS and HS. Totally 342 DEGs were found through pairwise comparisons with 158 DEGs in LS and 24 in HS when compared with CS group. The top significantly DEGs in the low and high salinity indicate that the improper salinity environment might cause metabolic disorders. From the most significantly expressed genes in LS and HS including *GBDL*, *PCFT*, *ESULT*, *RDL1*, *NKTASα*, *CTL1*, *NKCC*, *TRET1*, *HcS2*, *VTGL*, *CK2α*, *calponin/transgelin*, *ZFRBL*, *MAP 1AL*, *APNL*, *mucin-1*, *NEP11L, MRP1*, *PRJ*, *ZFRB*, *SMC2* and *GILT3*, it indicated that in the short-term salinity changes, the megalopa will regulate some mechanisms such as metabolism, oxidative stress, osmoregulation to adapt the alteration of the environment. Thus, the salinity change might impact the growth by megalopa and cause immunity responses. The top five significantly enriched KEGG pathway by DEGs in LS and HS were mostly related to immunity responses. These pathways and gene expression patterns provide new insights into the molecular mechanisms of immune responses and osmotic regulation under salinity stress in crustaceans.

## Methods

### Ethics statement

All experiments were conducted with strict accordance with the recommendations in the Guide for the Care and use of Laboratory Animals of Shantou University and approval of the Institutional Animal Care and Use Ethics Committee of Shantou University. All efforts were made to minimize *S. paramamosain* suffering.

### Sources of megalopa, experimental design and sampling

The experiment was conducted at Raoping West Coast Biotechnology Co. Ltd., Chaozhou, Guangdong province, China. Females *S. paramamosain* with full ovarian development (ovarian maturation Stage V) [88] were purchased from local fishermen in May 2019. Crabs were held in 20 m^3^ cement pond with disinfected seawater (temperature: 25–30 °C; salinity: 26–29 ppt) and fed once daily in the afternoon (3:00 pm) with razor clam *Sinonovacula constricta* at approximately 10% body weight. Before spawning, the berried crabs were reared individually. When the color of eggs turned to dark grey or black, the berried crab was transferred to a cement pond (5 × 4 × 1.5 m). The newly hatched larvae were cultured at a density of 50–100 individuals per liter and were fed with rotifer, *Brachionus* sp., at Zoea I and Zoea II stage. When the larvae metamorphose to Zoea III, the diet was switched to brine shrimp *Artemia nauplii*. The salinity and water temperature were maintained between 26 and 29 ppt, 25 and 30 °C, respectively.

A preliminary experiment was set six levels of salinity, namely 7, 14, 21, 28, 35 and 42 ppt to determine the optimum salinity of the mud crab megalopa. One day after zoea metamorphosed to megalopa, the megalopa with robust vitality were selected out for salinity treatment. The metamorphosis number of the megalopa was recorded when the megalopa begin to metamorphose. After 8 days, the metamorphosis rate of each group was calculated respectively. Based on the metamorphosis rate of megalopa under different salinities in the preliminary experiment, the salinities were set as 14, 25 and 39 ppt with triplicates. One hundred robust megalopa were utilized for each replicate cultured in 50 L culture tanks. The megalopa were collected according to the timeline at 0, 12, 24, 48 and 72 h, stored in RNAlater (Vazyme BioTechnologies Co., Ltd., Nanjing, Jiangsu, China) for subsequent RNA extraction. The megalopa were fed adult Artemia in both experiments. The mean temperature was 28 ± 2 °C for the experiment.

### RNA isolation and sequencing

Total RNA was extracted from megalopa treated in 14, 25 and 39 ppt salinities for 24 h, respectively. Each treatment was set three replicates and each replicate used eight individuals for RNA isolation using the Trizol kit (Invitrogen, USA) following the manufacturer’s instructions. The purity, concentration and integrity of RNA samples are tested using NanoDrop 2000 (Thermo Scientific, Waltham, MA) and Agilent 2100 Bioanalyzer (Agilent, Santa Clara, CA) to ensure qualified samples prior to transcriptome sequencing.

Totally 1 μg of qualified RNA (RNA quality score (RQS) 6.0–8.0 and the OD260/280 1.8–2.0) per group was directed at RNA sample preparations. Libraries for sequencing were generated using NEBNext UltraTM RNA Library Prep Kit for Illumina (NEB, USA) according to manufacturer’s instructions. Subsequently, each sample attached with index codes to characterize sequences. In brief, mRNAs were purified from total RNA using poly T oligomer magnetic beads. In NEBNext First-Strand Synthesis Reaction Buffer (5X), bivalent cation is used for cleavage at high temperature. The first strand cDNA was synthesized by random hexamer primers and M-MuLV Reverse Transcriptase. The second strand of cDNA was then synthesized using DNA Polymerase I and RNase H, and the remaining overhangs were converted to blunt end by exonuclease/polymerase. After the 3 ‘end of DNA fragment was adenylated, the NEBNext adaptor of hairpin loop structure was ligated for hybridization. To preferentially select 240 bp cDNA fragments, library fragments were purified using the AMPure XP system (Beckman Co., Beverly, USA). The USER enzyme (NEB, USA) was added to the cDNA with selected size and adaptor, and PCR was performed with Phusion High-fidelity DNA polymerase after 15 min at 37 °C and 5 min at 95 °C, universal PCR primers and index (X) primers were used for PCR. Finally, the PCR products were purified by AMPure XP system and the quality of the library was evaluated on the Agilent Bioanalyzer 2100 system. According to the manufacturer’s suggestion, TruSeq PE Cluster Kit v4-cBot -HS (Illumia) was used to cluster the index-coded samples on the cBot Cluster Generation System. After clustered, library preparations were sequenced on Illumina platform to generate paired-end reads by Biomarker BioTechnologies Co. Ltd., Beijing, China.

### Data analysis and functional annotation

Raw data (raw reads) generated by Sequencing By Synthesis technology in fastq format were first processed by an in house perl scripts which is proprietary by Biomarker BioTechnologies Co. Ltd., Beijing, China. In this step, clean data (clean reads) could be obtained by removing the reads that contained the adaptor or ploy-N and the low-quality reads in the raw data. Meanwhile, the Q20, Q30, GC-content and sequence repetition levels of clean data were calculated. All downstream analyses were based on high quality clean data. These clean reads were subsequently hit to reference genome sequences (unpublished data, the genome is available upon request.). Only reads with maximum one mismatch were further analyzed and annotated against the reference genome. HISAT2 software ([46], http://ccb.jhu.edu/software/hisat2/index.shtml) were used to map with the reference genome.

Gene function was annotated based on the following databases: Nr (NCBI non-redundant protein sequences, ftp://ftp.ncbi.nih.gov/blast/db/), Swiss-Prot (A manually annotated and reviewed protein sequence database, http://www.uniprot.org/), Pfam (Protein family, http://pfam.xfam.org/), KOG (The database of Clusters of Protein homology, http://www.ncbi.nlm.nih.gov/KOG/), GO (Gene Ontology, http://www.geneontology.org/) and KEGG (The database of Kyoto Encyclopedia of Genes and Genomes, http://www.genome.jp/kegg/).

### Quantification of gene expression levels and differential expression analysis

Gene expression levels were calculated by fragments per kilobase of transcription per million (FPKM). DESeq2 was used for differential expression analysis between every two groups and provided statistical routines to determine differential expression of digital gene expression data using a model based on negative binomial distribution. The *P* value was adjusted using methods of Benjamini and Hochberg to control the false discovery rate. Genes with *p* value less than 0.01 was considered as differentially expressed gene (DEGs). The GO enrichment analysis of DEGs was performed using GOseq R software packages based on the Wallenius non-central hypergeometric distribution [92], which can adjust the gene length bias in DEGs. KEGG [43] is a database resources to understand the advanced features and practical application of biological systems, such as cells, biological and ecological system, the information from the molecular level, especially large-scale genome sequencing of molecular data sets and other high-throughput experimental technologies (http://www.genome.jp/kegg/). We used KOBAS 2.0 [89] to test the statistical enrichment of DEGs in the KEGG pathway.

### Quantitative real-time PCR for RNA-seq results validation

Nine genes were screened out for RNA-seq validation using quantitative real-time PCR (qRT-PCR) with Talent qPCR Premix (SYBR Green) kit (TIANGEN Biotech Co., Ltd., Beijing) according to the manufacturer’s instructions. cDNA was produced from 2 μg total RNA using FastKing gDNA Dispelling RT Supermix (TIANGEN Biotech Co., Ltd., Beijing). Primers (supplementary Table 1) for qRT-PCR were designed using the Primer 6.0 Software. qRT-PCR was performed in a Mini Option real-time detector (Roche Light Cycle@480). The qRT-PCR reaction solution included 5 μl Talent qPCR Premix (2×) (TIANGEN Biotech Co., Ltd., Beijing), 0.4 μl PCR forward primer (10 μM), 0.4 μl PCR reverse primer (10 μM), 2.0 μl cDNA solution (20 ng), and 2.2 μl RNase-free water. The reaction conditions were followed by the recommendation of the instruction. Agarose gel electrophoresis was performed on all amplifiers to determine their size. The expression level of each gene was normalized towards the reference gene (*18 s rRNA*). The optimized comparative Ct (2^-ΔΔCt^) value method was applied here to calculate gene expression levels.

## Supplementary information

**Additional file 1: Supplementary Fig. 1.** The species distribution of the result of NR annotation.

**Additional file 2: Supplementary Fig. 2.** KOG function classification of consensus sequences of all the transcripts detected in RNA-Seq of *Scylla paramamosain* megalopa under different levels of salinities.

**Additional file 3: Supplementary Fig. 3.** Gene ontology (GO) assignment of assembled transcripts of *Scylla paramamosain* megalopa.

**Additional file 4: Supplementary Table 1.** Primers used for qRT-PCR. **Supplementary Table 2.** Summary of RNA sequencing of the *Scylla paramamosain* megalopa under different salinities. **Supplementary Table 3.** Differentially expressed genes in the comparison of low salinity (LS) and control salinity (CS) in *Scylla paramamosain* megalopa. **Supplementary Table 4.** Differentially expressed genes in the comparison of high salinity (HS) and control salinity (CS) in *Scylla paramamosain* megalopa.

## Data Availability

The datasets generated during the current study were deposited in the National Center for Biotechnology Information (NCBI) Sequence Read Archive (SRA, http://www.ncbi.nlm.nih.gov/Traces/sra) with the accession numbers SRR10493620, SRR10493619 and SRR10493618 corresponding to the LS, CS and HS group, respectively.
