# Supplementary figures and images for: mRNA profile provides novel insights into stress adaptation in mud crab megalopa, Scylla paramamosain after salinity stress
Source: BMC Genomics. 2020 Aug 14;21:559. doi: 10.1186/s12864-020-06965-5 (PMC7430823; doi:10.1186/s12864-020-06965-5)

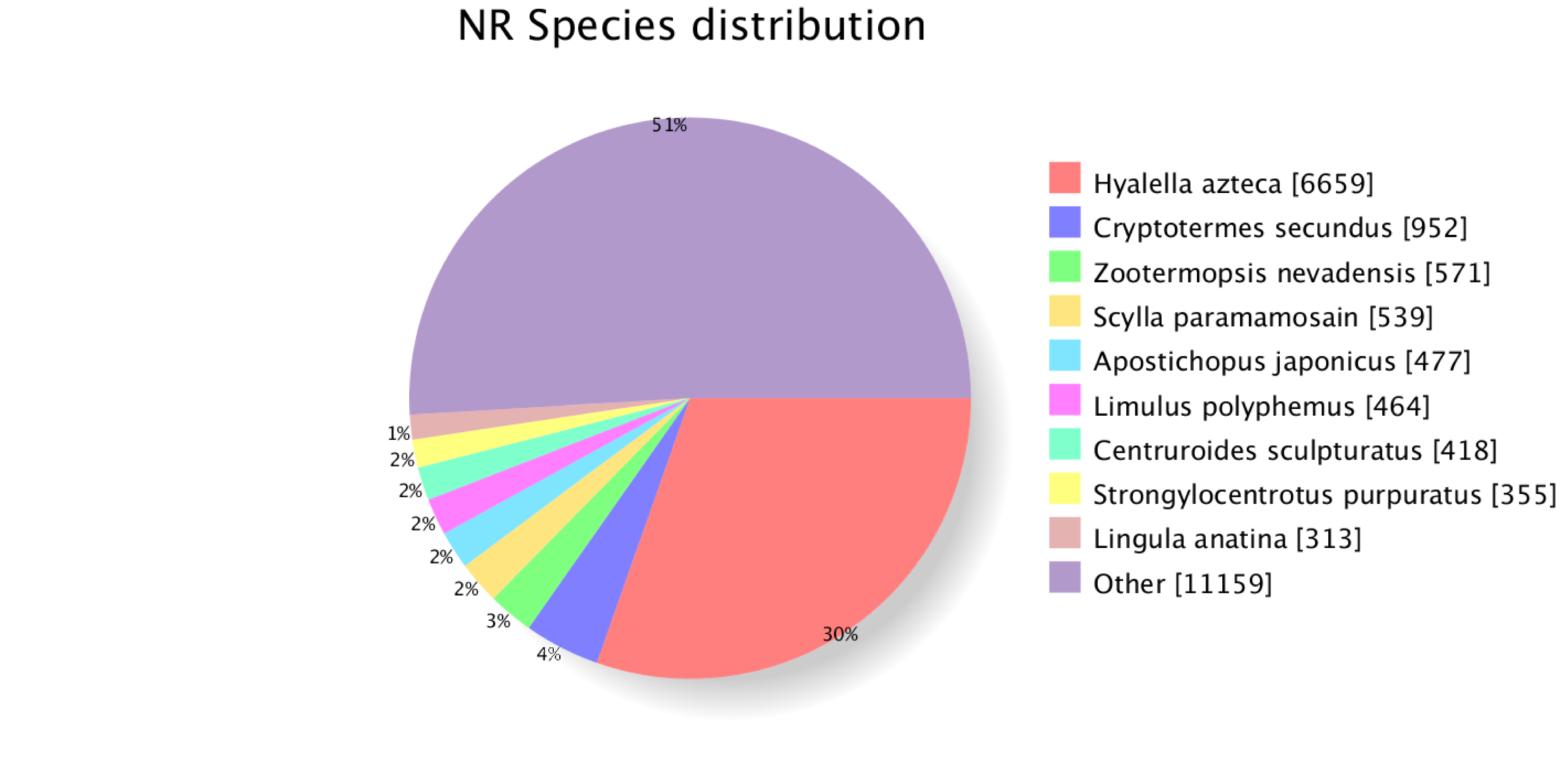

Supplement: Supplementary file 1 — Additional file 1: Supplementary Fig. 1. The species distribution of the result of NR annotation. [file 12864_2020_6965_MOESM1_ESM.tif]

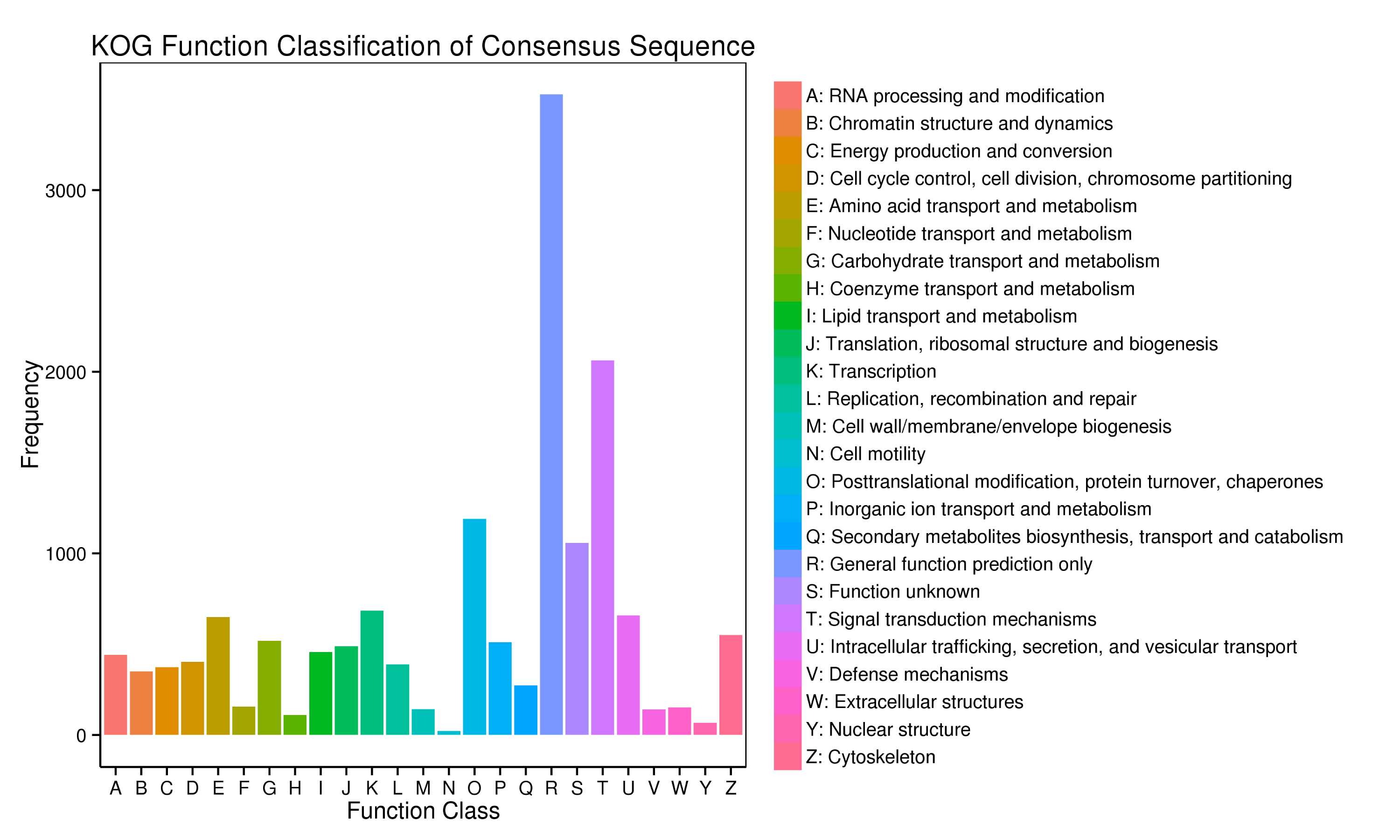

Supplement: Supplementary file 2 — Additional file 2: Supplementary Fig. 2. KOG function classification of consensus sequences of all the transcripts detected in RNA-Seq of Scylla paramamosain megalopa under different levels of salinities. [file 12864_2020_6965_MOESM2_ESM.tif]

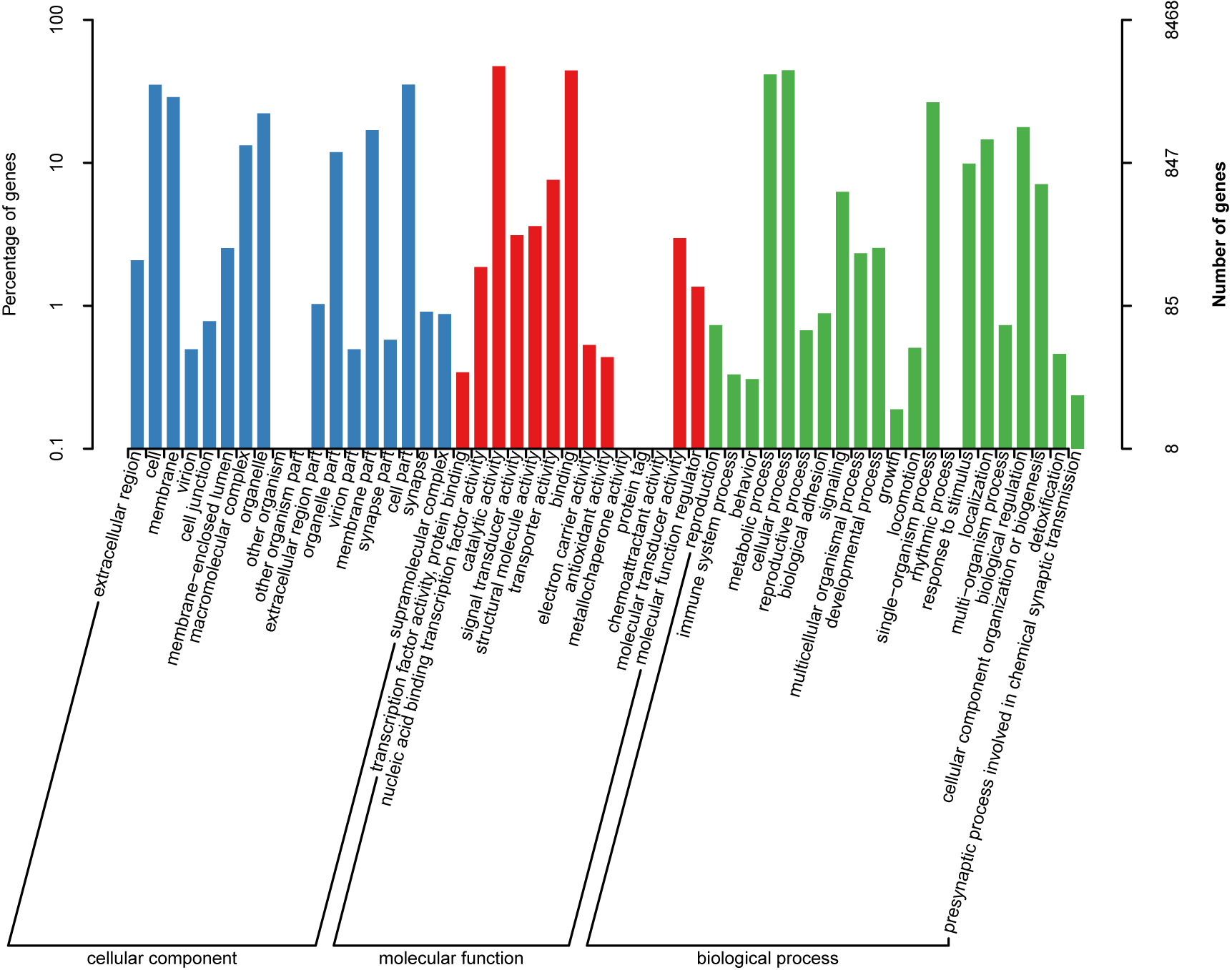

Supplement: Supplementary file 3 — Additional file 3: Supplementary Fig. 3. Gene ontology (GO) assignment of assembled transcripts of Scylla paramamosain megalopa. [file 12864_2020_6965_MOESM3_ESM.tif]
